# The Enlargement of Abdominal Lymph Nodes Is a Characteristic of Autoimmune Liver Disease

**DOI:** 10.1155/2020/3631625

**Published:** 2020-03-21

**Authors:** Yongjuan Wang, Xiuxiu Xu, Maojuan Ran, Xiaopei Guo, Lu Zhou, Xi Wang, Bangmao Wang, Jie Zhang

**Affiliations:** ^1^Department of Gastroenterology and Hepatology, General Hospital, Tianjin Medical University, Tianjin Institute of Digestive Disease, Tianjin 300052, China; ^2^Department of Gastroenterology and Hepatology, The Second Affiliated Hospital of Hebei Medical University, Hebei, China

## Abstract

**Background:**

The enlargement of lymph nodes is a common clinical sign in connective tissue disease (CTD) and viral hepatitis. In this research, we evaluated the incidence of enlarged lymph nodes in autoimmune liver diseases (AILD). Moreover, we identified the clinical significance of abdominal lymph node enlargement in AILD.

**Methods:**

The characteristics of abdominal lymph nodes, including their morphology and distribution, were assessed by ultrasonography and computed tomography in 125 patients with AILD, 54 with viral hepatitis, 135 with CTD, and 80 healthy controls. The pathological and laboratory results of 106 AILD patients were collected to analyze the association between lymphadenectasis and disease activity.

**Results:**

Enlargement of abdominal lymph nodes was found in 69.6% of patients with AILD, 63% of patients with viral hepatitis, 29.6% of patients with CTD, and 2% of healthy controls. Alkaline phosphatase (ALP), glutamate transpeptidase (GGT), and immunoglobulin M (IgM) levels were significantly increased in AILD patients with lymphadenectasis (LA) in contrast to patients without lymphadenectasis (NLA) (*P* < 0.05). The pathological characteristics of inflammation, cholestasis, and focal necrosis were more common in the LA group than in the NLA group (*P* < 0.05). As shown by multivariate logistic regression analysis, interface hepatitis (OR = 3.651, *P* < 0.05), cholestasis (OR = 8.137, *P* < 0.05), and focal necrosis (OR = 5.212, *P* < 0.05) were related to LA.

**Conclusions:**

The percentage of abdominal lymph node enlargement in AILD subjects was significantly higher than that in CTD subjects. Therefore, the enlargement of lymph nodes can represent a noninvasive indicator of histological and biochemical inflammation activity in AILD.

## 1. Introduction

Autoimmune liver disease (AILD) is a common cause of chronic hepatitis that leads to liver cirrhosis due to occult onset [1]. The categories of AILD include autoimmune hepatitis (AIH), primary biliary cholangitis (PBC), primary sclerotic cholangitis (PSC), and overlap syndrome. At present, AILD remains a major diagnostic and therapeutic challenge due to the lower incidence of disease and heterogeneous subtypes [2, 3].

Inflammatory response in organs usually leads to hyperplasia of regional lymph nodes. Enlarged abdominal lymph nodes are a common finding in patients with chronic active hepatitis [4, 5], especially in those caused by autoimmune [6, 7] or viral infection [8–10]. In addition, a higher incidence of enlarged abdominal lymph nodes in PBC (74–100%) and AIH (13–73%) has been reported [6].

The existing research shows that the enlargement of lymph nodes in multiple parts of the body is a shared clinical manifestation in connective tissue diseases (CTD) [11, 12]. CTD comprises a group of immune system diseases involving the connective tissues of the body. Patients with CTD can have positive antinuclear antibodies (ANA) and increased IgG levels, which can also be found in AILD patients [11]. Furthermore, it has been reported that enlarged lymph nodes are associated with disease activity in CTD. Researchers also found that enlarged abdominal lymph nodes in chronic hepatitis C (CHC) subjects are associated with serum parameters of viremia, a high frequency of serum CD8 levels, and severe histological damage [13, 14]. However, the characteristics of enlarged lymph nodes in CTD, CHC, and AILD have not been studied. In addition, the association between the enlargement of lymph nodes and AILD activity is still unclear.

We speculated that lymphoid hyperplasia was the response of an altered immune system to an undefined antigenic stimulus. In the present study, we analyzed the incidence of enlarged lymph nodes in CTD, viral hepatitis, and AILD. Then, we evaluated their association with disease activity by comparing them with biochemical, immunological, and pathological results in AILD subjects. In addition, we assessed the distribution of abdominal lymph nodes in CTD, viral hepatitis, and three subtypes of AILD. The results indicated that the enlargement of lymph nodes is a noninvasive indicator of histological and biochemical inflammation activity in AILD.

## 2. Methods

### 2.1. Patients

For the study, 225 individuals with AILD were recruited from October 2008 to May 2016. The diagnosis of AILD was made according to EASL guidelines (AIH), AASLD guidelines (PBC), and the Paris standard (AIH-PBC) [15, 16]. All patients were negative for neoplasm, lymphadenoma, and intestinal tuberculosis. Exclusion criteria were applied, with the following results: 46 patients were excluded for a history of viral hepatitis, alcoholic liver disease, drug-induced liver disease, or CTD; 54 patients were excluded for incomplete data. Ultimately, 125 AILD subjects were enrolled in the study, 106 of whom underwent liver biopsies. Additionally, 135 patients with CTD, as diagnosed by the American College of Rheumatology (ACR) and the European League Against Rheumatism (EULAR) [17–19], were enrolled in the study from October 2008 to May 2016. All patients were negative for a history of neoplasm, lymphadenoma, intestinal tuberculosis, and multifarious liver diseases. Moreover, 54 patients with viral hepatitis, as diagnosed by EASL guidelines, were also investigated [20, 21]. As a control group, 80 healthy volunteers were recruited for the study. The study was approved by the ethics committee of Tianjin Medical University General Hospital. All patients and control volunteers were at least 18 years old and provided informed consent to participate in the study.

### 2.2. Serologic and Pathological Laboratory Tests

Laboratory tests were performed on AILD subjects: alanine amino transferase (ALT), aspartate amino transferase (AST), alkaline phosphatase (ALP), glutamate transpeptidase (GGT), and total bilirubin (TBIL); IgG, IgM, and IgA; and anti-nuclear antibody (ANA), smooth muscle antibody (SMA), anti-liver kidney microsomal antibody (Anti-LKM), anti-mitochondrial antibody (AMA), and anti-mitochondrial antibody 2 (AMA2). All the above-mentioned parameters were detected before treatment. Within the lobular parenchyma, the following morphological changes were evaluated: hepatocyte edema, interface hepatitis, focal necrosis, and hepatic fibrosis. All liver biopsy specimens were checked independently by two experienced pathologists.

### 2.3. Computed Tomography Image Analysis

Celiac lymph nodes of the liver and abdominal organs were assessed for echotexture by ultrasonography and computed tomography. Abdominal lymph nodes larger than 5 mm in the shortest diameter were counted, and the size of each lymph node was measured in the longest axis (a) and in the corresponding perpendicular axis (b) using ALOKA ultrasound and TSX-032A computed tomography, respectively. Examinations were performed by two experienced radiologists, and patients with consistent evaluation results were included in the study. The maximum cross-sectional area was recorded as the result after multiple measurements.

### 2.4. Statistics

Statistical analysis was completed with SPSS 7.5 for Windows (SPSS Inc., Chicago, IL, USA). Demographic characteristics of patients were expressed as median range or mean ± SD. Statistical significance between two groups of normally distributed quantitative data was analyzed by a *t*-test. The Mann-Whitney *U* test was used for categorical and nonnormal continuous data. For qualitative data, comparisons among the groups were conducted using the *χ*^2^ test or Fischer's exact test. Multivariate logistic regression analysis was used to identify factors that were independently associated with the presence of abdominal lymph nodes. A *P* value < 0.05 was considered statistically significant.

## 3. Results

### 3.1. The Frequency of Abdominal Lymph Nodes in AILD Is Higher Than That in CTD

**Figure 1:**
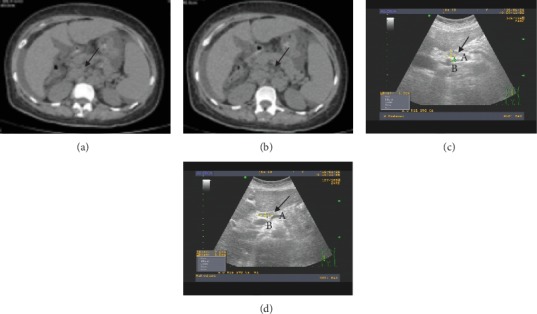
Enlarged lymph node (LN) was detected in autoimmune liver disease (AILD). (a) Abdominal CT examination indicated the enlargement of para-aortic lymph nodes. (b) Abdominal CT examination showed the enlargement of pancreatic lymph nodes. (c) Abdominal ultrasonography showed round enlarged lymph nodes around the pancreas. (d) Abdominal ultrasonography revealed oval enlarged lymph nodes around the pancreas. Abdominal lymph nodes larger than 5 mm but at their shortest diameter were counted. The arrow points to the lymph node: (A) longest axis and (B) perpendicular axis.

As shown in Table 1, the baseline demographic data of all the patients were listed, and the frequency of abdominal lymph nodes was detected (Figure 1). The proportion of gender at the time of the study was the only demographic parameter which differed significantly among all the groups (*P* < 0.05). Enlarged lymph nodes occurred in all groups, and a significantly higher proportion of lymph nodes was found in AILD subjects than in control group subjects (*P* < 0.001). Abdominal lymph nodes tended to be more prevalent in patients with AILD (69.6%) and viral hepatitis (63%), whereas enlarged lymph nodes were only infrequently observed in patients with CTD and in control patients (29% and 2%, respectively). The positive proposition of abdominal lymph node enlargement in the subjects with AIH, PBC, and overlap syndrome was 56%, 86.2%, and 73.9%, respectively (*P* = 0.014), while the positive rate of lymph node enlargement in PBC subjects was significantly higher than that in AIH subjects (*P* = 0.006).

### 3.2. The Enlargement of Abdominal Lymph Nodes Is Positively Correlated with Disease Activities in AILD

**Figure 2:**
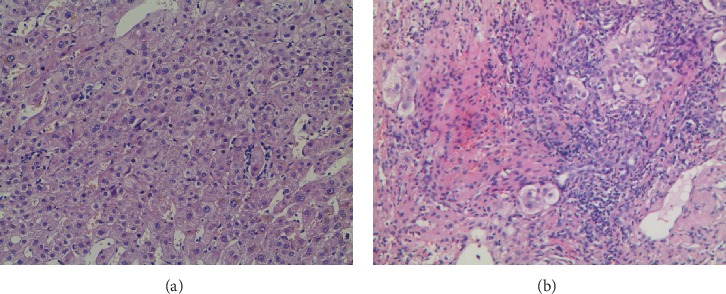
Typical liver biopsy from patients with or without lymph node enlargement. (a) In patients with lymph node enlargement, leukomonocyte infiltrates and focal necrosis are present. (b) In patients with lymph node enlargement, interface hepatitis, leukomonocyte infiltrates, cholestasis, focal necrosis, and hepatic rosette formation are present.

As shown in Table 2, there were no significant differences in age or in the proportion of females in the LA group and NLA group. In addition, the levels of ALP and GGT in the LA group were higher than those in the NLA group: 181 U/L (126 U/L, 367 U/L) vs. 132 U/L (92.8 U/L, 166 U/L); 199 U/L (124 U/L, 416 U/L) vs. 104.5 U/L (55.3 U/L, 180 U/L), *P* < 0.05, respectively; meanwhile, there were no significant differences in other liver function test results. The patients in the LA group were observed to have a higher level of IgM (294 mg/dL (137 mg/dL, 474 mg/dL) vs. 247.5 mg/dL (98.8 mg/dL, 361 mg/dL)) than those in the NLA group (*P* < 0.05). It was also observed that interface hepatitis occurred in 22.2% of patients without lymph node enlargement, whereas up to 48.6% of those with lymph node enlargement presented with interface hepatitis (*P* = 0.009). Cholestasis was found in 30% of LA group subjects and 5.6% of NLA group subjects (*P* = 0.004), and focal necrosis was found in 31.4% of LA group subjects and 5.6% of NLA group subjects (*P* = 0.003). Meanwhile, no statistical significance in terms of inflammation of the portal area and hepatic fibrosis was found (*P* > 0.05). It was indicated that histological damage, including interface hepatitis, focal necrosis, and cholestasis, was more severe in the LA group than in the NLA group (*P* < 0.05). As liver biopsy specimens showed, patients with lymph node enlargement presented with interface hepatitis, leukomonocyte infiltrates, cholestasis, focal necrosis, and hepatic rosette formation (Figure 2(b)), whereas patients without lymph node enlargement only presented with leukomonocyte infiltrates and focal necrosis (Figure 2(a)).

The correlations between biochemical and pathological characteristics and lymph nodes in AILD were assessed (Table 3). The enlargement of lymph nodes was not found to be correlated with serum levels of AST (*P* = 0.468), ALP (*P* = 0.337), or GGT (*P* = 0.167). In contrast, significant positive associations were observed between lymph nodes and interface hepatitis (*P* = 0.019), cholestasis (*P* = 0.011), and focal necrosis (*P* = 0.044).

The volume of abdominal lymph nodes in AILD was greater than that in viral hepatitis.

The morphological characteristics of lymph nodes, which have a circular or oval shape with clear boundaries and no fusion phenomena, can be detected by abdominal ultrasound and abdominal CT (Figure 1). The size of lymph nodes in AILD patients was found to be larger in comparison with that of lymph nodes in viral hepatitis patients. As shown in Table 4, ultrasound testing revealed that abdominal lymphadenopathy appeared in 50 of 87 (57.5%) cases of AILD and 21 of 34 (61.8%) cases of viral hepatitis. The number of abdominal lymph nodes was more than 1 in 46 cases (92%) of AILD patients and 19 cases (90.5%) of viral hepatitis patients, which was not significant (*P* = 0.999). Similarly, the aspect ratio (*a*/*b*) of lymph nodes greater than 2 presented in 13 cases (26%) of AILD and 6 cases (28.6%) of viral hepatitis, and there was no significant difference between the two groups (*P* = 0.823). Moreover, the maximum cross-sectional area of lymph nodes in AILD patients was larger than that in patients with viral hepatitis (1.34 cm^2^ (0.82 cm^2^, 2.07 cm^2^) vs. 0.96 cm^2^ (0.68 cm^2^, 1.32 cm^2^), *P* = 0.017).

### 3.3. The Distribution of Lymph Nodes in AILD Was More Common in the Periphery of the Pancreas and Porta Hepatis, Especially in Patients with AIH

The distribution of lymph nodes in different liver injury diseases was determined (Table 5). In AILD, the frequencies of lymphadenopathy in the periphery of the pancreas, porta hepatis, abdominal aorta, gastrohepatic ligament, and mesenteric roots were 37.9%, 33.3%, 32.2%, 17.2%, and 2.3%, respectively, while in CTD patients, these frequencies were 5%, 17.5%, 85%, 10%, and 47.5%, respectively. Abdominal lymph nodes appeared more often in the periphery of the pancreas (*P* < 0.001) and porta hepatis (*P* = 0.014) in AILD patients than in patients with CTD, while abdominal lymph nodes were commonly found in the abdominal aorta and mesenteric roots in CTD patients (*P* < 0.001). Compared to viral hepatitis, in AILD, lymph nodes occurred mainly in the periphery of the pancreas (*P* = 0.005) and gastrohepatic ligament (*P* = 0.006).

The distributions of lymph nodes in different subtypes of AILD were also analyzed (Table 5). In patients with AIH, the frequencies of lymphadenopathy in the periphery of the pancreas, porta hepatis, abdominal aorta, gastrohepatic ligament, and mesenteric roots were 53.6%, 50%, 25%, 10.7%, and 0%, respectively, while in PBC patients, these frequencies were 5%, 17.5%, 85%, 10%, and 47.5%, respectively (Table 5). In patients with AIH-PBC, the frequencies of lymphadenopathy in the periphery of the pancreas, porta hepatis, abdominal aorta, gastrohepatic ligament, and mesenteric roots were 41.2%, 17.6%, 26.5%, 14.7%, and 0%, respectively. Abdominal lymph nodes mainly appeared in the periphery of the pancreas in AIH and AIH-PBC; meanwhile, in PBC, lymph nodes were mainly found in the abdominal aorta (Table 5).

## 4. Discussion

One study showed a higher incidence of enlarged lymph nodes in PBC (74–100%) and AIH (13–73%) [6]. In addition, abdominal lymph node enlargement could be the only imaging manifestation in patients with AILD [14]. It was reported that lymph nodes in patients with CTD were not limited to the body surface, but were in fact also distributed in the abdominal cavity [22]. However, in our study, abdominal lymph nodes tended to be most prevalent in patients with AILD (69.6%), significantly higher than in CTD patients (29.6%). We speculated that lymphoid hyperplasia was the response of an altered immune system to an undefined antigenic stimulus. It was reported that the lymph nodes in CTD were more commonly found on the body surface, which may be because CTD is an immune system disease involving connective tissues [23, 24]. The enlargement of lymph nodes in AILD was mainly found in the abdominal cavity, since AILD is an immune-related liver disease [6].

The sizes of the noticeable lymph nodes seemed to be histologically and serologically correlated with disease activity in patients with AILD [14]. The enlargement of perihepatic lymph nodes in chronic hepatitis C was shown to be related to liver histology and hepatitis C virus viremia, which in turn reflects the inflammatory activities and immunological responses of the host [25]. Previous studies have shown that enlarged regional lymph nodes are significantly correlated with the elevation of ALP and GGT, which is more common in chronic liver diseases [26]. In our study, the levels of ALP and GGT were significantly higher in the LA group than in the NLA group, which might indicate the persistence of inflammation. Several studies have suggested that regional lymph node enlargement is significantly correlated with ALT, AST, ALP, serum bilirubin, serum anti-mitochondrial antibodies, and IgG, reflecting hepatocellular damage, cholestasis, and humoral immunoreactivity in PBC. The increase of ALT and AST indicated that inflammatory damage to the liver persists; likewise, the increase of ALP and serum bilirubin indicated inflammatory damage to the bile duct [13, 26]. In our research, abdominal lymphadenopathy in subjects with AILD was related to histopathological severity, including interface inflammation, focal necrosis, and cholestasis. This study showed that patients with abdominal lymph node enlargement should undergo liver biopsy to analyze the activity and severity of liver inflammation and that timely treatment should be considered in these patients.

Nakanishi et al. observed enlarged lymph nodes in 77–91% of patients with CHC and 96% of patients with CHB [27]. In our study, abdominal lymph nodes tended to be prevalent both in patients with AILD (69.6%) and in those with viral hepatitis. The ultrasound results showed that the size of lymph nodes in subjects with AILD was larger than that in subjects with viral hepatitis. This finding is important, as it might contribute to the identification of AILD and viral hepatitis.

It has been demonstrated that swelling of the mediastinal lymph node may be involved in patients with rheumatoid arthritis (RA), systemic lupus erythematosus (SLE), and mixed connective tissue disease (MCTD) [28]. The distribution of lymph nodes was extensive in SLE, including the axillary, cervical, supraclavicular, and inguinal regions; in RA, however, a lymph node often occurred in the lymphatic drainage of involved joints. In our study, abdominal lymph nodes were frequently found in the periphery of the pancreas and porta hepatis in patients with AILD; in contrast, abdominal lymph nodes were more common in the para-aorta and mesenteric roots in patients with CTD.

In our study, the distribution of lymph nodes in different subtypes of AILD was analyzed as well. The abdominal lymph nodes mainly appeared in the periphery of the pancreas and porta hepatis in AIH patients (*P* < 0.05); in patients with PBC, abdominal lymph nodes were more commonly found in the abdominal aorta. It has been reported that pancreatic lymph nodes can be divided into two groups—a pancreatic head group and the posterior wall of the pancreatic head group—and can both be considered “interchange stations” in the abdominal lymphatic system between the hepatic lymph nodes and the mesenteric lymph nodes [29]. We speculate that lymph nodes play a vital role in the pathogenesis of AIH at the periphery of the pancreas and porta hepatis. Efe et al. reported that the proportion of PBC patients with AIH features was high in an extended follow-up period [29]. This study showed that when pancreatic lymph nodes were found in PBC patients, these patients appeared to have AIH features as well. Therefore, the pathogenesis of different subtypes of AILD and abdominal lymph nodes warrants further research.

There were several limitations to our study. First, errors may have occurred because even though 106 of 125 individuals underwent liver biopsy and subsequent assessment of pathological features, this number of cases has little statistical significance. We also did not attempt to correlate disease activity with actual nodal size, only with lymph node enlargement.

In conclusion, the enlargement of perihepatic lymph nodes in AILD subjects can act as a good indicator, one that reflects the histological and biochemical inflammatory activities of the liver. Abdominal lymph nodes mainly appeared in the periphery of the pancreas in patients with AILD, while in CTD and viral hepatitis patients, abdominal lymph nodes frequently occurred in the abdominal aorta and mesenteric roots. Future studies with larger groups of patients are needed to further analyze the effects and mechanisms of abdominal lymph nodes in AILD. Toward this end, we will explore whether changes in the size of lymph nodes can predict a sustained response of AILD therapy.

## Figures and Tables

**Table 1:** Baseline demographics and positive rates of abdominal lymphadenopathy in patients and healthy controls.

Diseases	Number	F/M	Mean age (y)	Patients with lymphadenopathy	*P*
AILD	125	(114/11)	59 ± 0.99	87 (69.6%)	—
AIH	50	(45/5)	62 ± 1.46	28 (56%)	
PBC	29	(28/1)	59.1 ± 1.8	25 (86.2%)	0.006^∗∗^
AIH-PBC	46	(41/5)	58 ± 1.6	34 (73.9%)	
Viral hepatitis	54	(28/26)	55 ± 2.54	34 (63%)	0.306^∗^
CTD	135	(103/32)	52.5 ± 2.38	40 (29.6%)	<0.001^∗^
Healthy	80	(48/32)	48 ± 6.27	1 (2%)	<0.001^∗^

The positive rate of abdominal lymph node enlargement in different groups was compared by the *χ*^2^ test. ^∗^The AILD group was compared with the viral hepatitis, CTD, and health groups, respectively, and *P* < 0.05 was statistically significant. ^∗∗^The PBC group was compared with the AIH group. AILD: autoimmune liver disease; AIH: autoimmune hepatitis; PBC: primary biliary cholangitis; AIH-PBC: overlap syndrome; CTD: connective tissue diseases.

**Table 2:** Univariate analysis of demographic, biochemical, immunological, and pathological characteristics in 106 cases of AILD subjects.

	LA (*n* = 70)	NLA (*n* = 36)	*P*
Demography			
Age (years)	59.67 ± 1.3	58.53 ± 1.9	0.609
Female	64 (91.4%)	33 (91.7%)	0.999
Biochemistry			
ALT (U/L)	72 (36, 154)	57 (31, 146.25)	0.620^∗^
AST (U/L)	81 (41, 136)	50.5 (28.75, 110)	0.073^∗^
ALP (U/L)	181 (126, 367)	132 (92.8, 166)	0.001^∗^
GGT (U/L)	199 (124, 416)	104.5 (55.3, 180)	0.000^∗^
TBIL (mol/L)	20.5 (13.1, 47.6)	19.8 (11.6, 31.9)	0.334^∗^
Immunology IgG (mg/dL)	1610 (1360, 1840)	1620 (1337, 1875)	0.942^∗^
IgM (mg/dL)	294 (137, 474)	247.5 (98.8, 361)	0.035^∗^
IgA (mg/dL)	327 (233, 454)	298 (228.8, 405)	0.733^∗^
Histopathology			
Portal area inflammation	58 (82.9%)	26 (72.2%)	0.201^∗∗^
Interface hepatitis	34 (48.6%)	8 (22.2%)	0.009^∗∗^
Cholestasis	21 (30%)	2 (5.6%)	0.004^∗∗^
Focal necrosis	22 (31.4%)	2 (5.6%)	0.003^∗∗^

The data are presented as medians and quartiles. ^∗^The analysis of the biochemical parameters in the LA and NLA groups in AILD was performed by the Mann-Whitney test. ^∗∗^The analysis of the pathological parameters in the LA and NLA groups in AILD was conducted by the *χ*^2^ test, with *P* < 0.05 being considered statistically significant. AILD: autoimmune liver disease; LA: lymphadenectasis; NLA: nonlymphadenectasis; ALT: alanine amino transferase; AST: aspartate amino transferase; ALP: alkaline phosphatase; GGT: glutamate transpeptidase; TBIL: total bilirubin.

**Table 3:** Factors associated with the presence of abdominal lymph nodes in patients with AILD: multivariate analysis.

Variable	Odds ratio (95% CI)	^∗^ *P*
AST (IU/L)	1.001 (0.998, 1.004)	0.468
ALP (IU/L)	1.002 (0.998, 1.007)	0.337
GGT (IU/L)	1.003 (0.999, 1.007)	0.167
Interface hepatitis	3.651 (1.231, 10.3)	0.019
Cholestasis	8.137 (1.606, 41.232)	0.011
Focal necrosis	5.212 (1.046, 25.96)	0.044

^∗^Analysis of the correlation between biochemical and pathological indexes and LN in AILD using logistic regression. The data are presented as medians and quartiles. *P* < 0.05 was considered statistically significant. AILD: autoimmune liver disease; AST: aspartate amino transferase; ALP: alkaline phosphatase; GGT: glutamate transpeptidase.

**Table 4:** The morphological characteristics of lymph nodes in patients with AILD and viral hepatitis.

Features	AILD	Viral hepatitis	*P*
	(*n* = 50)	(*n* = 21)	
Number			
=1	4 (8%)	2 (9.5%)	0.999^∗^
>1	46 (92%)	19 (90.5%)	
Aspect ratio (*a*/*b*)			
<2	37 (74%)	15 (71.4%)	0.823^∗^
≥2	13 (26%)	6 (28.6%)	
The maximum cross-sectionalarea of LN (cm^2^)	1.34 (0.82, 2.07)	0.96 (0.68, 1.32)	0.017^∗∗^

Number: lymph node number. Aspect ratio: longest axis (*a*)/perpendicular axis (*b*). Maximum cross-sectional area of LN: *a* × *b*. ^∗^The analysis of the number and aspect ratio of LN between AILD and viral hepatitis was completed by the *χ*^2^ test. ^∗∗^The analysis of the maximum cross-sectional area of LN between AILD and viral hepatitis was performed using the *F* test. *P* < 0.05 was considered statistically significant. AILD: autoimmune liver disease; LN: lymph nodes.

**Table 5:** Frequency of lymphadenopathy detected in various organs in patients with AILD, CTD, and viral hepatitis.

Disease	Pancreatic	Hepatic	Para-aorta	Hepatogastric	Mesentery
	*N* (%)	*N* (%)	*N* (%)	*N* (%)	*N* (%)
AILD (*n* = 87)	33 (37.9)	29 (33.3)	28 (32.2)	15 (17.2)	2 (2.3)
AIH (*n* = 28)	15 (53.6)	14 (50)	7 (25)	3 (10.7)	0 (0)
PBC (*n* = 25)	4 (16)	9 (36)	12 (48)	7 (28)	2 (8)
AIH-PBC (*n* = 34)	14 (41.2)	6 (17.6)	9 (26.5)	5 (14.7)	0 (0)
CTD (*n* = 40)	2 (5)	7 (17.5)	34 (85)	4 (10)	19 (47.5)
Viral hepatitis (*n* = 34)	4 (11.8)	10 (29.4)	25 (73.5)	14 (41.2)	17 (50)

^∗^ *P* < 0.05 was considered statistically significant. AILD: autoimmune liver disease; AIH: autoimmune hepatitis; PBC: primary biliary cholangitis; AIH-PBC: overlap syndrome; CTD: connective tissue diseases.

## Data Availability

All the data are available at the correspondence author upon request.
